# Novel *IKBKG* gene mutations in incontinentia pigmenti: report of two cases

**DOI:** 10.3389/fmed.2023.1303590

**Published:** 2023-12-19

**Authors:** Huaqing Chen, Xiaojuan Ji, Yun Lai, Ling Xie, Chunlei Wan, Longnian Li

**Affiliations:** Department of Dermatology and Venereology, Candidate Branch of National Clinical Research Centre for Skin and Immune Diseases, The First Affiliated Hospital of Gannan Medical University, Ganzhou, China

**Keywords:** incontinentia pigmenti, *IKBKG* gene, gene mutation, genotype–phenotype correlation, gene detection

## Abstract

Incontinentia pigmenti (IP), an X-chromosome dominant genodermatosis caused by mutations in the *IKBKG*/*NEMO* gene, is a rare disease affecting the skin, teeth, eyes, and central nervous system. Here, we report two pedigrees of IP and detection of two novel mutations in the *IKBKG* gene associated with IP via genetic analysis. In addition, different gene mutation types can present with different clinical phenotypes, and the same gene mutation type can show different clinical phenotypes. This study provides clinical cases for further study of the genotype and phenotype of IP and enriches the mutation spectrum of *IKBKG* gene, which provides a basis for genetic counseling and genetic diagnosis of IP in the future.

## 1 Introduction

Incontinentia pigmenti (IP) is a rare, X-chromosome dominant genodermatosis caused by mutations in the *IKBKG*/*NEMO* gene that affects the skin, teeth, eyes, and central nervous system (1). The typical skin lesions of IP begin in infancy and go through four stages, with occasional overlap: erythema and vesicle, verrucous hyperplasia, hyperpigmentation following Blaschko’s line distribution, and linear and atrophic hypopigmented. In most patients, several stages often overlap or lack a certain stage (2). Most patients also present with eosinophilia in the skin and blood in the first stage (3). The diagnosis of IP is primarily based on characteristic skin lesions, histological examination, and genetic analysis (3). However, any condition with skin manifestations within Blaschko’s lines may be confused with IP, including different types of epidermolysis bullosa, X-linked-dominant chondrodysplasia punctata and linear epidermal nevi, Naegeli syndrome, and different types of ectodermal dysplasia. The age of the patient and the onset of changes are very important. Differential diagnosis in the context of skin changes depends on the IP stage (3). If there are no visible skin changes, the differential diagnosis is far more difficult, especially if no other family members have received a diagnosis of IP. For all of these reasons and with the development of genetic testing technology, genetic analysis is becoming increasingly important for the diagnosis of IP (4). Here, we report two cases of IP with novel mutations in the *IKBKG* gene.

## 2 Case report

**Figure 1:**
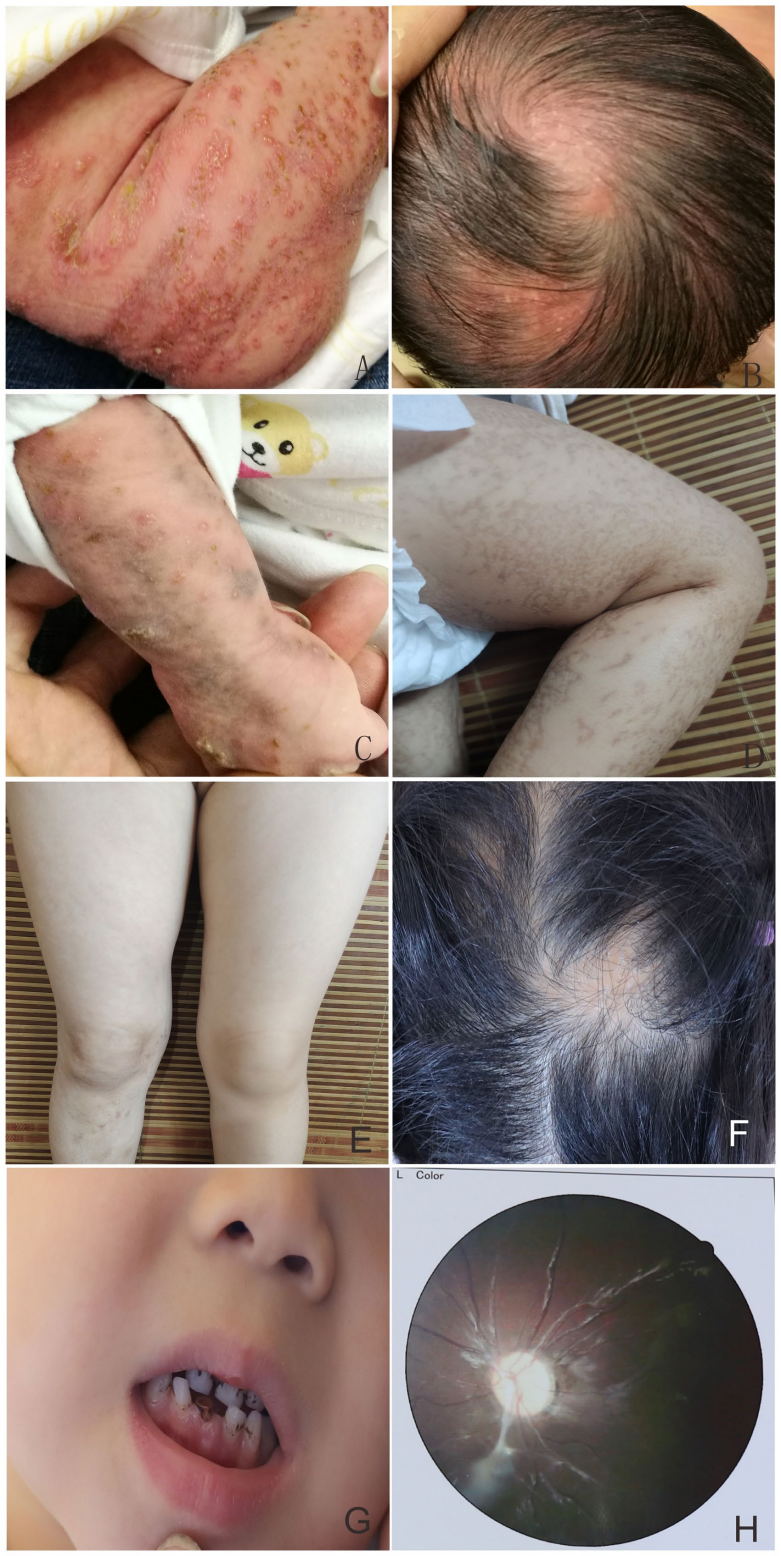
Linear erythema and blisters on the trunk, limbs, and scalp presented in the proband **(A,B)**. At 1.5 months, multiple typical skin lesions overlapped with linear erythema and blisters, verrucous hyperplasia, and hyperpigmentation **(C)**. Hyperpigmented lesions in the proband at 5 months of age **(D)**. Minor linear hyperpigmentation and hypopigmentation in both lower limbs **(E)**, focal alopecia on the head **(F)**, hypoplastic teeth **(G)**, optic atrophy in the left eye **(H)** at 4 years of age.

**Figure 2:**
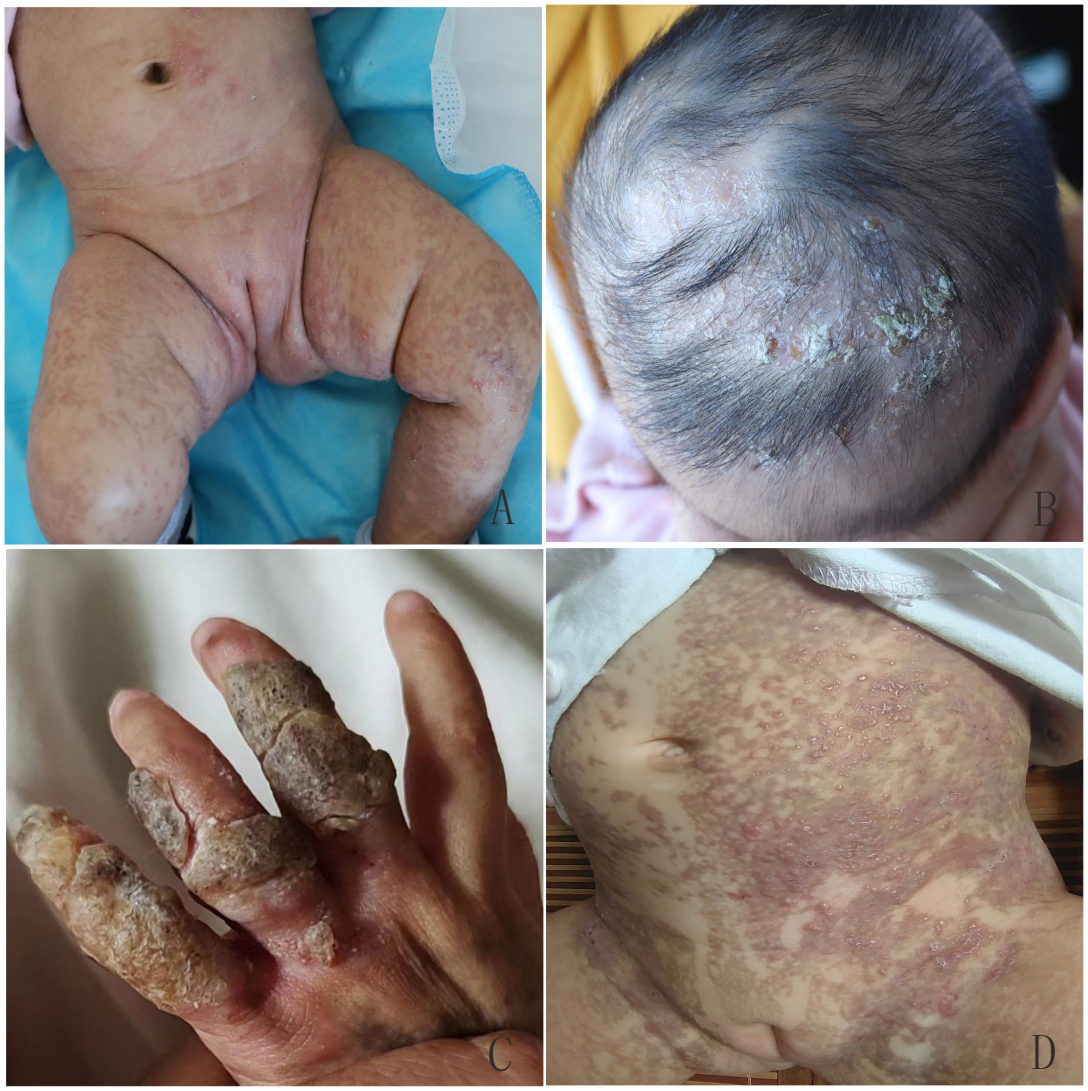
Overlapping skin lesions of erythema and blisters, verrucous hyperplasia, and pigmentation on proband’s sister **(A)**, and verrucous hyperplasia and local alopecia on the scalp **(B)** at 1.5 months of age. Verrucous hyperplasia on the fingers **(C)** and recurrent erythema and blisters on the trunk **(D)** at 10 months of age.

**Figure 3:**
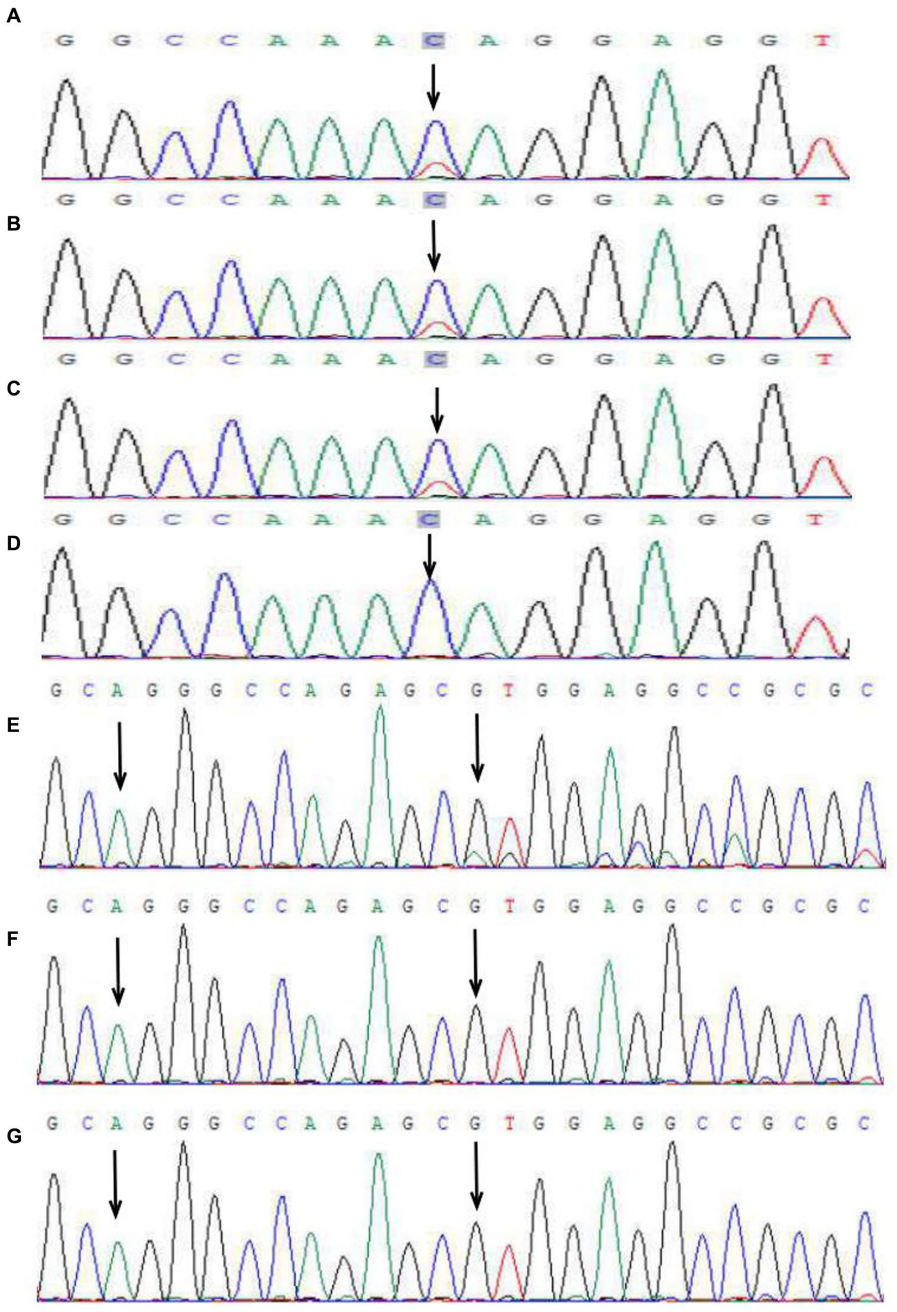
Peak map of gene mutation sequencing in two families. In the first family, *IKBKG* c.832C>T in the proband **(A)**, her sister **(B)**, and her mother **(C)** (black arrow). No mutation at the same locus was detected in the father **(D)** (black arrow). A base repeat mutation *IKBKG* c.614_624dup was detected in the patient of the second family (the first arrow), and the repeat sequence (AGGGCCAGAGC) was shown (start from the second arrow), which generated frameshift mutation, namely, c.614_624dup (p.Val209Argfs*76) **(E)**. No mutation was detected in the mother **(F)** and grandmother **(G)** in the second family.

In the first family, the proband was a newborn girl who had developed linear erythema and blisters on the trunk, limbs, and scalp after birth (Figures 1A,B). At 1.5 months, three typical skin lesions were observed: erythema and blisters, verrucous hyperplasia, and overlapping hyperpigmentation (Figure 1C). At 5 months of age, the lesions were predominantly linear hyperpigmentation, without erythema, blisters, or verrucous hyperplasia lesions (Figure 1D). When the proband was 4 years old, some clinical manifestations still existed, including small linear hyperpigmentation and hypopigmentation on both lower limbs, focal hair loss on the head, poorly developed teeth, optic atrophy in the left eye, and myopia (Figures 1E–H). In September 2022, the proband’s 1.5 months-old sister was admitted to the hospital with skin lesions similar to those of the proband (Figures 2A,B). However, at 10 months of age, she still had prominent verrucous hyperplastic lesions on her fingers, recurrent erythema, and blisters on the trunk (Figures 2C,D). Their mothers also had a similar rash at birth, but the symptoms were mild, and no skin lesions or other IP-associated symptoms were detected. All other family members were healthy. After obtaining informed consent, genomic DNA was extracted from peripheral blood of the proband, her younger sister, and her parents. Whole exome sequencing, MLPA, and Sanger sequencing of the *IKBKG* gene were performed. The mutation c.832C>T (p.Gln278*) in exon 6 of the *IKBKG* gene was detected in the proband, her sister, and her mother, but not in her father (Figures 3A–D).

**Figure 4:**
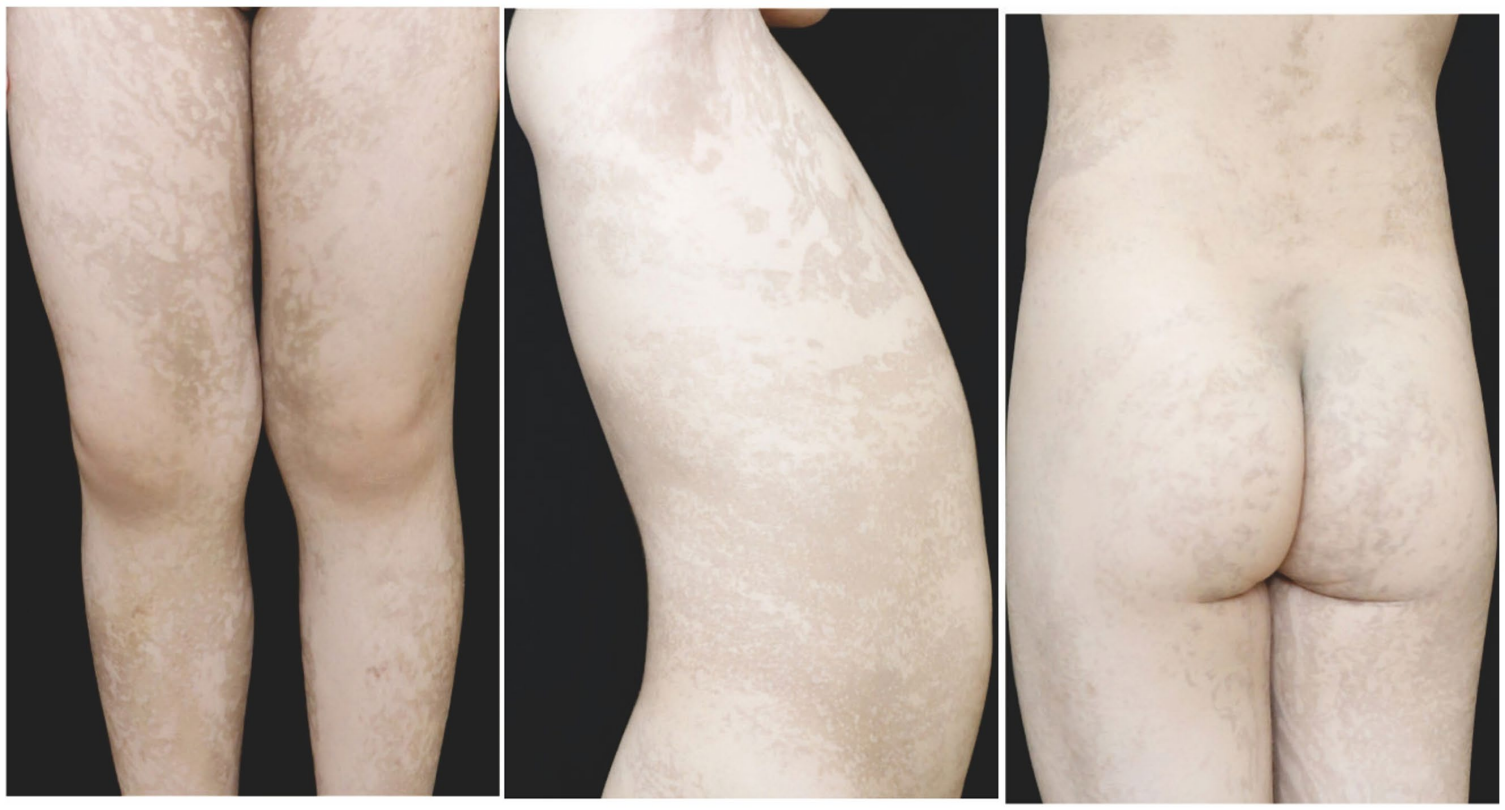
At 4 years old, the patient in the second family presented with linear hyperpigmentation on the trunk and limbs.

In the second family, the patient was a 4 years-old girl with linear hyperpigmentation of the trunk and limbs distributed along Blaschko’s lines (Figure 4). No abnormalities were found in the nails, hair, teeth, eyes, or central nervous system. Erythema and blisters were detected on the trunk and limbs after birth, and the skin lesions gradually progressed to linear pigmentation within 3 months. The patient’s parents and other family members were healthy. After obtaining informed consent, genomic DNA was extracted from the peripheral blood. Whole exome sequencing, MLPA and Sanger sequencing of *IKBKG* gene were performed. Mutation c.614_624dup (p.Val209Argfs*76) in exon 5 of the *IKBKG* gene was detected in the patient, and no relevant gene mutations were detected in her mother or grandmother (Figures 3E–G).

IP was diagnosed based on typical skin lesions and *IKBKG* gene mutations (3). No specific treatment was administered. During the 4 years follow-up period, the skin symptoms continued to improve, and only a small amount of linear hyperpigmentation and hypopigmentation was observed in both lower limbs of the proband. However, focal alopecia, hypoplastic teeth, optic atrophy in the left eye, and myopia persisted (Figures 1E–H). A topical steroid cream was prescribed to improve the prominent verrucous hyperplasia lesions on the fingers of the proband’s sister in the first family. After 1 year, obvious verrucous hyperplastic lesions remained on the sister’s fingers, and recurrent erythema and blisters on her trunk (Figures 2C,D), focal alopecia, and a suspicious retinal lesion in the right eye (Figure 2B) were observed. During the follow-up period, no abnormal central nervous system manifestations were observed in the first family member. The patient in the second family did not receive any special treatment because she only had hyperpigmented skin lesions at initial presentation. During the 1 year follow-up, no abnormal manifestations of the nails, hair, teeth, eyes, or central nervous system were observed in the family members.

## 3 Discussion

IP is caused by mutations in *IKBKG*, resulting in a loss of function. The most common mutation was the deletion of exons 4–10 in the *IKBKG* gene. Other mutation types include missense, frameshift, nonsense, and splice site change (5). The *IKBKG* gene encodes NEMO/IKKgamma, a regulatory protein of nuclear factor kappaB (NF-κB) signal transduction. NF-κB plays an important role in regulating cell proliferation, apoptosis, and inflammation. When *IKBKG* is not correctly expressed, cells become sensitive to apoptotic signals, leading to the development of various inflammatory responses that are particularly active in ectodermal cells (6).

Studies have shown that deletions of exons 4–10 and most minor mutations in the *IKBKG* gene are loss-of-function mutations, resulting in an IP phenotype of extreme X chromosome skewing and inactivation in females and fetal death in males (5). The mutations *IKBKG* c.832C>T (p.Gln278*) and *IKBKG* c.614_624dup (p.Val209Argfs*76) can cause the stop codon to appear prematurely, which lead to the loss or defect of *IKBKG* protein, resulting in the loss of the normal function or dysregulation of the NF-κB pathway, and finally produce the phenotype of IP.

At present, there are few relevant studies on the causes of IP recurrence, and it has been reported that some *IKBKG*-deficient keratinocytes can survive through escape; however, residual *IKBKG*-deficient keratinocytes that manage to escape and survive the elimination process can undergo second episodes of the first stage of IP owing to the reoccurrence of keratinocyte hyperproliferation and subsequent inflammatory reactions (6, 7). The vesicular phase usually disappears within four to 6 months (2). However, the sister of the proband in the first family had repeated episodes of erythema and blisters on the trunk throughout almost one full year of follow-up, which may be related to residual mutant keratinocyte hyperproliferation and inflammation. Previous studies have shown that ocular abnormalities are often accompanied by neurological manifestations and determine the severity of IP (8). Therefore, we recommend close follow-up for the progression of IP-related diseases and appropriate imaging studies as a preventive measure. Particularly in patients with recurrence, close follow-up is necessary.

There are many molecular diagnostic methods, the most common of which are whole-exon sequencing, MLPA detection, simple PCR, and Sanger sequencing (7). Owing to the existence of the pseudogene *IKBKGP1*, multiple methods are sometimes used for genetic testing (6). No mutation was detected in whole exome sequencing, and MLPA detection and Sanger sequencing were performed to identify the mutation. Therefore, it is important to select the appropriate genetic testing for detecting gene mutations. Molecular diagnosis is helpful for the diagnosis of IP, for a greater understanding of the genotype–phenotype correlation of IP, and for clinicians to guide patients and their families in counseling about prognosis and future reproductive options (7).

Few studies have examined the genotype–phenotype correlation, and some studies have shown that there is no obvious correlation between the genotype and phenotype of IP (7–10). The gene mutation type of the proband in the first family differed from that of the patient in the second family, and the clinical manifestations of the proband were more severe than those of the patient in the second family. However, this proband had the same genetic mutation as her sister and mother, and their clinical manifestations differed. The phenotypic heterogeneity of IP is attributed to skewed X chromosome inactivation and the propensity for selection against cells in which the normal X chromosome is inactivated (11). The specific reasons for this need to be further studied, such as by observing and counting the genotypic and phenotypic characteristics of many clinical cases. These two pedigree studies will be helpful for further studies on the correlation between the IP phenotype and genotype.

Owing to its rarity and highly variable phenotypic expression, IP may remain an undiagnosed systemic disease, sometimes with a very high psychological, physical, economic, and social burden on patients and their families. As there is no curative treatment, early diagnosis of IP based on skin features, histopathological examination, and genetic testing is important for genetic counseling and timely therapeutic interventions.

## 4 Conclusion

We detected two novel mutations of *IKBKG* gene associated with IP, which enriched the mutation spectrum of *IKBKG* gene. It is helpful to further define the relationship between *IKBKG* mutations and IP, study the phenotype–genotype correlation of IP, and help clinicians guide the counseling of patients and their families regarding prognosis and future reproductive options.

## Data availability statement

The datasets presented in this article are not readily available because of ethical/privacy restrictions. Requests to access the datasets should be directed to the corresponding authors.

## Ethics statement

The studies involving humans were approved by The First Affiliated Hospital of Gannan Medical University. The studies were conducted in accordance with the local legislation and institutional requirements. Written informed consent for participation in this study was provided by the participants’ legal guardians/next of kin. Written informed consent was obtained from the individual(s), and minor(s)’ legal guardian/next of kin, for the publication of any potentially identifiable images or data included in this article.

## Author contributions

HC: Conceptualization, Data curation, Investigation, Methodology, Project administration, Writing – original draft, Writing – review & editing. XJ: Data curation, Formal analysis, Methodology, Project administration, Software, Writing – original draft, Writing – review & editing. YL: Software, Writing – review & editing, Conceptualization, Investigation, Visualization. LX: Data curation, Methodology, Software, Visualization, Writing – review & editing. CW: Conceptualization, Funding acquisition, Methodology, Resources, Supervision, Writing – original draft, Writing – review & editing. LL: Conceptualization, Writing – review & editing, Funding acquisition, Methodology, Resources, Supervision, Writing – original draft.
